# Syphilis-related rapidly progressive glomerulonephritis: a case presentation

**DOI:** 10.1186/s12882-021-02404-z

**Published:** 2021-05-25

**Authors:** A. Qi, P. O. Fiset, L. Pilozzi-Edmonds

**Affiliations:** 1 Department of Nephrology, St-Mary’s Hospital, McGill University, 3830 av Lacombe, Qc H3T 1M5 Montreal, Canada; 2 Department of Pathology, McGill University, Montreal, QC Canada

**Keywords:** Syphilis, Rapidly progressive glomerulonephritis, Case report, Proteinuria, Hematuria

## Abstract

**Background:**

Syphilis is a multisystemic infection that causes a wide variety of symptoms and thus has been dubbed one of the great medical mimickers. Due to recent global re-emergence of syphilis, it has become important to recognize its various presentations. Relative to the kidney, syphilitic infections generally present themselves with nephrotic range proteinuria, and are most often associated with pathological features of a membranous glomerulonephritis with subepithelial immune complex deposition. However, other rare renal presentations have been reported. One of these includes a rapidly progressive glomerulonephritis picture. All described cases have been successfully resolved with the treatment of the underlying syphilis infection.

**Case presentation:**

The patient was an elderly woman of Caribbean descent who presented with lower extremity weakness, anasarca and proteinuria, hematuria with progressive renal failure. On kidney biopsy, she was found to have a pauci-immune crescentic glomerulonephritis pattern and a concomitant acute tubulointerstitial nephritis. She had a positive *Treponema pallidum* particle agglutination test and a negative syphilis rapid plasma reagin test with clinical evidence of polyneuropathy suggestive chronic syphilis infection.

**Conclusion and discussion:**

It is important in the context of pauci-immune crescentic glomerulonephritis to explore all differential diagnoses. Given the positive syphilis serologies, clinical context and presence of tubulointerstitial nephritis, she was determined to have syphilitic glomerulonephritis that resolved with a course of both penicillin and steroids.

## Background

Rapidly progressive glomerulonephritis (RPGN) is diagnosed when a patient presents with hematuria, proteinuria and increasing serum creatinine over a short period of time. The histopathologic correlate of RPGN is crescentic glomerulonephritis and is commonly classified based on three main patterns: type I is defined as anti-glomerular basement membrane (GBM) positive disease, type II is immune-complex mediated disease and type III is pauci-immune disease. Several infections lead to immunologic dysregulations that result in type II RPGN. The most common infections being post-streptococcal glomerulonephritis caused by streptococcal infection and membranoproliferative glomerulonephritis caused by viral infections such as hepatitis. However, a much rarer cause is syphilis, with only three reported cases [1–3]. Generally, syphilitic infection has been associated with nephrotic-range proteinuria caused by membranous glomerulonephritis [4–6]. As there is a global rise in syphilis cases, it is important to recognize atypical syphilitic presentations, especially if there is a pattern of RPGN. We present a case of a patient with proteinuria, hematuria and progressive renal failure compatible with a pattern of RPGN.

## Case presentation

A retired 77-year-old Caribbean woman presented to the emergency department with general fatigue and lower extremity weakness. She is known for type II diabetes mellitus, hypertension, and a recent episode of small bowel obstruction that had resolved without surgery.

On examination, she had a bilateral severe axonal sensory and motor polyneuropathy (confirmed by nerve conduction studies and electromyography) which was manifested by severe bilateral lower extremity weakness. She also had diffuse anasarca. She did not have any skin findings nor any ophthalmologic symptoms.

She had a previously normal creatinine of 70 μmol/L (0.79 mg/dL), but on admission was found to have an acute kidney injury with a serum creatinine of 122 μmol/L (1.38 mg/dL). The creatinine peaked over 3 weeks to 231 μmol/L (2.61 mg/dL) (Table 1). This was associated with a decreasing albumin and increasing urine protein (Table 1). Urinalysis showed significant proteinuria and hematuria and urine microscopy showed active sediments including dysmorphic erythrocytes. Urine eosinophils were positive twice. A serological panel, which included cytoplasmic and perinuclear anti-neutrophilic antibodies anti-cytoplasmic (p-ANCA and c-ANCA), complement (C3 and C4), anti-nuclear antibodies (ANA), hepatitis B, hepatitis C and human immunodeficiency virus (HIV) serologies were all negative (Table 1). Rheumatoid factor (RF), C-reactive protein (CRP) and the immunoglobulin profile (IMM) were consistent with inflammation. Both the urine protein electrophoresis and the serum protein electrophoresis were normal. Given the neurological symptoms and impressive proteinuria, a Venereal Disease Research Laboratory (VDRL) and *Treponema pallidum* particle agglutination test (TP-PA) were requested and both were positive. A peripheral electromyography (EMG) with nerve conduction studies, was also consistent with syphilis-induced neuropathy. The patient declined a lumbar puncture to confirm neurosyphilis and confirmed she had never been diagnosed with nor specifically been treated for syphilis before.

**Table 1:** Relevant Laboratory Data

Laboratory Test	Admission (Day 0)	Renal Consultation (Day 30)	Day of Biopsy (Day 37)	1-week post-treatment initiation (Day 44)	1-month post-treatment (Day 74)	1 year Post-treatment
Serum creatinine (μmol/L)	122	157	231	133	110	97
Albumin (g/L)	21	14	29 (on IV albumin)	18	25	35
Urine protein (g/g)	0.48	1.28	1.22		0.54	0.83
ANCA				Negative		
ANA			Negative			
Complement			Normal			
IMM Profile			Inflammatory		Normal	
IgG (g/L)			19.6		15.6	
IgA (g/L)			5.35		4.56	
IgM (g/L)			0.68		0.86	
CRP (mg/L)			173.4		14	
RF			Positive			
Creatine kinase (IU/L)			9			
Hepatitis B			Negative			
Hepatitis C			Negative			
HIV			Negative			
Syphilis			Syphilis total IgG and IgM reactive VDRL (+) TPPA (+) RPR (−)		Syphilis total IgG and IgM reactive VDRL (+) TPPA (+) RPR (−)	

**Fig. 1:**
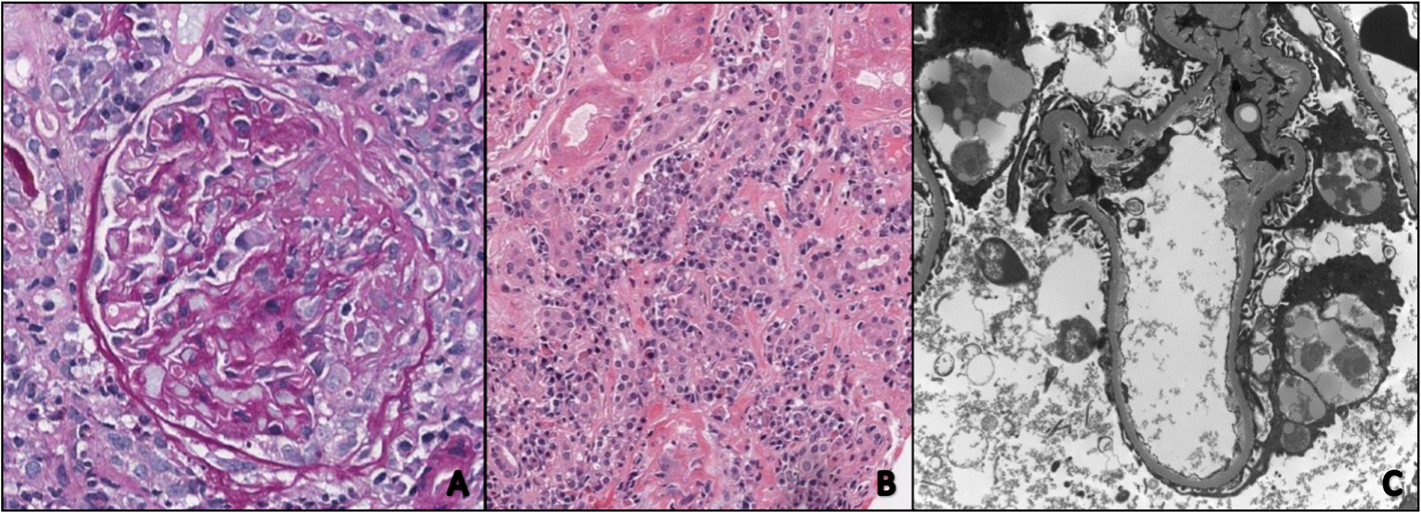
**a** Light microscopy of kidney biopsy showing crescent formation in the glomerulus. Periodic Acid-Schiff stain (400X). **b** Light microscopy showing tubulointerstitial nephritis with large infiltration of plasma cells and eosinophils. Haematoxylin and Eosin stain (200X) (**c**) Electron microscopy did not show any significant immune complex deposits (3000X)

**Fig. 2:**
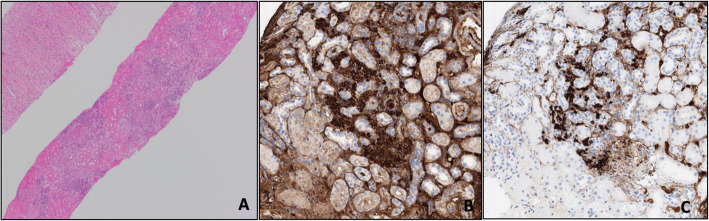
**a** Light microscopy of the kidney biopsy core with significant interstitial plasma cell and eosinophil infiltration. H&E 40X. **b** IgG Immunohistochemistry staining. **c** IgG4 subclass immunohistochemisty staining (accounting for ~ 50% of IgG staining)

While waiting for the serological testing, a kidney biopsy was performed due to the increasing creatinine with an active urine sediment. The biopsy showed crescentic and necrotizing glomerulonephritis in 9/23 glomeruli (Fig. 1a). Some of the crescentic glomeruli even showed Bowman’s capsule rupture. Numerous plasma cells surrounded the crescentic glomeruli and some of the small blood vessels. There was also concomitant acute tubulo-interstitial nephritis with abundant plasma cells and occasional eosinophils (Figs. 1 and 2). The plasma cells showed an increased proportion (50%) of IgG-4 versus IgG (Fig. 2). There was moderate interstitial fibrosis and tubular atrophy as well as moderate atherosclerosis. No granulomatous inflammation was identified. Glomerular basement spikes or vacuolization were not found. No *Treponema pallidum* were found by immunohistochemistry and there was no evidence for monoclonality by kappa/lambda in situ hybridization. Immunofluorescence showed only minimal focal non-specific C3 mesangial staining (1+) and fibrinogen positive crescents (3+). IgA, IgG, IgM, C1q, kappa and lambda immunofluorescence were negative. Electron microscopy did not show any significant electron dense deposits in any of the glomerular compartments (Fig. 1c).

Overall, the case was diagnosed as a pauci-immune crescentic glomerulonephritis with a concomitant acute tubulointerstitial nephritis. This was combined with atypical perivascular and periglomerular nonclonal, but significantly positive IgG-4 plasma cells infiltrates. The histological differential diagnosis would include a pauci-immune vasculitis, an atypical post-infectious glomerulonephritis, a secondary drug reaction or inflammatory reaction. Background vascular findings were attributed to the patient’s diabetes type 2 and hypertension. Our biopsy findings being similar to Nandikanti et al. [1] and given the clinical context, this presentation was most likely due to syphilitic infection. Absence of hypocomplementemia and of other organ involvement (thoraco-abdominal and pelvic computerized tomography showed no abnormalities) made IgG4-related disease and tubulointerstitial nephritis unlikely based on the proposed diagnostic criteria for IgG4-related disease [7, 8]. Furthermore, IgG4-disease with a lymphoplasmacytic pattern could not be diagnosed in the presence of an infection such as syphilis [9]. The immunoglobulin profile and RF were likely inflammatory given the C-reactive protein (CRP) of 174 mg/L. An elevated CRP is unlikely to be present IgG4-related disease [8]. The patient had a negative ANA and no symptoms of Sjogren’s syndrome and thus this diagnosis was excluded.

Given that we did not have the confirmatory syphilis test nor the ANCA serology test results initially and given this crescentic pauci-immune picture suggestive of ANCA vasculitis, we gave this elderly patient both intravenous (IV) penicillin G for 2 weeks and half-dose pulse methylprednisolone (500 mg IV daily X 3 days) concurrently. Once the ANCA results were negative, we continued with only the IV penicillin and a fast Prednisone taper. With this treatment, the patient’s serum creatinine decreased from a peak of 231 μmol/L (2.61 mg/dL) to 134 μmol/L (1.51 mg/dL) within 1 week. The serum creatinine remained at a new baseline of 110 μmol/L 1 month afterwards and 97 μmol/L a year later.

## Discussion and conclusions

We present a case of syphilitic RPGN in this patient with concomitant neurological symptoms due to neurosyphilis. As mentioned previously, RPGN typically presents in three broad immunohistopathological categories. It is important to consider the clinical context and send for the appropriate serological tests. In the case of syphilis, given the symptoms may be non-specific and mild, it is important to send a syphilis screen in the context of nephrotic range proteinuria and, as this case presentation and others have shown [1, 3], in the context of active urine sediment with hematuria and proteinuria. Timing is crucial, especially in crescentic-necrotizing renal disease - the earlier the diagnosis, the greater the chance of regression of crescent formation and thereby the greater the chance of renal recovery. In our case, the patient’s creatinine decreased in 2 days with treatment.

Similar to syphilitic membranous glomerulonephritis, management of syphilitic RPGN includes treating the underlying syphilitic infection [5, 6, 10–12]. As for immunosuppression with steroids, given the paucity of cases in literature of syphilitic RPGN, it is unknown whether steroids are beneficial for induction in the acute treatment phase. Two case reports have successfully treated with penicillin alone [1, 2]. Walker et al. treated a patient with RPGN who required dialysis with both penicillin and methylprednisolone [3]. We opted to give steroids given the cellular crescents and the pauci-immune appearance of the biopsy which was also suspicious for ANCA-associated RPGN. Intravenous penicillin was given for 14 days due to the suspicion of neurosyphilis. In brief, we believe it is prudent to consider steroids if the diagnosis is not clear, especially in the context of crescentic-necrotizing disease. However, the rapid and sustained response to penicillin and a short course of high dose steroids with rapid taper is not typical of an ANCA vasculitis and supports our diagnosis of syphilitic glomerulonephritis.

In conclusion, as the prevalence of syphilis increases, physicians should be mindful of the different renal manifestations of syphilitic infection. As latent syphilis may present vague symptoms or no symptoms, it is important to always consider latent syphilitic infection in any patient with isolated increasing proteinuria and any patient presenting with a RPGN pattern.

## Data Availability

Not applicable.

## Abbreviations

RPGN: Rapidly progressive glomerulonephritis
GBM: Anti-glomerular basement membrane
p-ANCA: Perinuclear anti-neutrophilic anti-cytoplasmic antibodies
c-ANCA: Cytoplasmic anti-neutrophilic anti-cytoplasmic antibodies
ANA: Anti-nuclear antibodies
IMM: Immunoglobulin profile
CRP: C-reactive protein
C3 and C4: Complement 3 and complement 4
RF: Rheumatoid factor
HIV: Human immunodeficiency virus
RPR: Rapid plasma reagin test
VDRL: Venereal Disease Research Laboratory
TP-PA: *Treponema pallidum* particle agglutination test
EMG: Electromyography
IV: Intravenous
H & E: Haematoxylin and eosin
